# Global historic pandemics caused by the FAM-1 genotype of *Phytophthora infestans* on six continents

**DOI:** 10.1038/s41598-021-90937-6

**Published:** 2021-06-11

**Authors:** Amanda C. Saville, Jean B. Ristaino

**Affiliations:** 1 Department of Entomology and Plant Pathology, North Carolina State University, 112 Derieux Place, Box 7613, Raleigh, NC USA; 2 Emerging Plant Disease and Global Food Security Cluster North, Carolina State University, 112 Derieux Place, Box 7613, Raleigh, NC USA

**Keywords:** Evolution, Plant sciences

## Abstract

The FAM-1 genotype of *Phytophthora infestans* caused late blight in the 1840s in the US and Europe and was responsible for the Irish famine. We sampled 140 herbarium specimens collected between 1845 and 1991 from six continents and used 12-plex microsatellite genotyping (SSR) to identify FAM-1 and the mtDNA lineage (Herb-1/Ia) present in historic samples. FAM-1 was detected in approximately 73% of the historic specimens and was found on six continents. The US-1 genotype was found later than FAM-1 on all continents except Australia/Oceania and in only 27% of the samples. FAM-1 was the first genotype detected in almost all the former British colonies from which samples were available. The data from historic outbreak samples suggest the FAM-1 genotype was widespread, diverse, and spread to Asia and Africa from European sources. The famine lineage spread to six continents over 144 years, remained widespread and likely spread during global colonization from Europe. In contrast, modern lineages of *P. infestans* are rapidly displaced and sexual recombination occurs in some regions.

## Introduction

Emerging plant diseases threaten crop production and forest ecosystems worldwide^1,2^. Movement of pathogens due to increased trade of plants and plant products has exacerbated outbreaks of plant diseases. *Phytophthora infestans* (Mont.) de Bary caused the Irish potato famine of the 1840s^2^, causes late blight on potato and tomato that threatens global food security worldwide^1^. While *P. infestans* can be spread aerially through asexual sporangia, long distance movement of the pathogen is mainly due to transport of infected tubers for use as seed potatoes^2^.

The history of *P. infestans* consists of a series of migrations, combined with periodic displacements of clonal lineages, both of which have occurred at local and global scales^2–4^. The disease first appeared in the US in 1843, near the ports of New York, Philadelphia and in surrounding states (Supplementary Table 1)^3,4^. The disease was first widely reported on the European continent two years later in Belgium in 1845, after which it spread throughout Europe and then into Ireland^2,3,5^. Others and we have studied historic populations of the pathogen in the US and Europe using mycological herbarium specimens to better understand the pathogen’s origin, identify the outbreak strain, and track its spread from the Americas to Europe^6–13^. Herbarium specimens collected in the 1840s and later from original outbreak specimens in the US and Europe revealed that the famine lineage was a Ia mitochondrial haplotype, disputing previous theories that the US-1 (Ib haplotype) genotype caused the famine^2,6,8,12–15^. The clonal lineage that caused the Irish potato famine was named FAM-1, the genome was sequenced, and its shared ancestry with *P. andina*, a sister species from South America, was documented^6,10,11^. The shared ancestry of *P andina* and the famine lineage suggest that both species may have been sympatric in South America^11^.

Microsatellite genotyping (SSRs) has also been used widely to study the population biology of *P. infestans*^2,14^. *P. infestans*-infected leaves from historic specimens collected in North America and Europe were genotyped using SSRs and migration from North America into Europe was documented^10^. The FAM-1 genotype eventually was displaced by the emerging US-1 genotype around the 1930s–1950s^9,10^. US-1 continued to persist globally until the 1980s, when it was replaced by more aggressive lineages out of Mexico and Europe, but can still be found in select populations today^2,10,15^.

Historic migrations of the pathogen into the US and Europe have been studied, but little is known about migrations of the FAM-1 genotype to other continents after the original nineteenth century outbreaks in the US and Europe^6,7,10–13^. Other researchers reported that the FAM-1 lineage was less fit and went extinct^8,9,16^. The earliest known observation of *P. infestans* on the Asian continent was in India between 1870 and 1880^17^, on the Australian/Oceania continent in Tasmania in 1907^4,18^, and in sub-Saharan Africa in Kenya in 1941^19^. More recent *P. infestans* lineages from the twenty-first century in east Africa were the US-1 genotype^20^ . The FAM-1 genotype was identified in Colombia, Guatemala, and Costa Rica between the 1910s and 1940s, suggesting that the FAM-1 genotype was present in Central and South America for many years^2,10^. First reports of the disease in Colombia were in 1861, in Peru in 1878 and in Mexico in 1898^2,4,21^. Herbarium samples of *P. infestans* from Solanaceous hosts in Central and South America are rare so actual dates of first reports are from available specimens and published sources^4,10,11^.

The goal of this study was to examine the global population structure of historic populations of *P. infestans* using a set of outbreak samples from mycological herbaria^22^. Our previous work focused on identifying the famine lineage and studying populations in the US and Europe^6,7,10^. The primary objectives of this research were: (1) to infer the population structure of historic *P. infestans* on six continents; (2) to examine the spatial and temporal biogeography and diversity of FAM-1 and US-1 genotypes; (3) to compare the impact of host diversity on genotype diversity; and (4) to infer migration pathways of the pathogen into Africa, Asia and the Australia/Oceania continents from putative US or European source populations.

## Results

### Population structure

**Figure 1:**
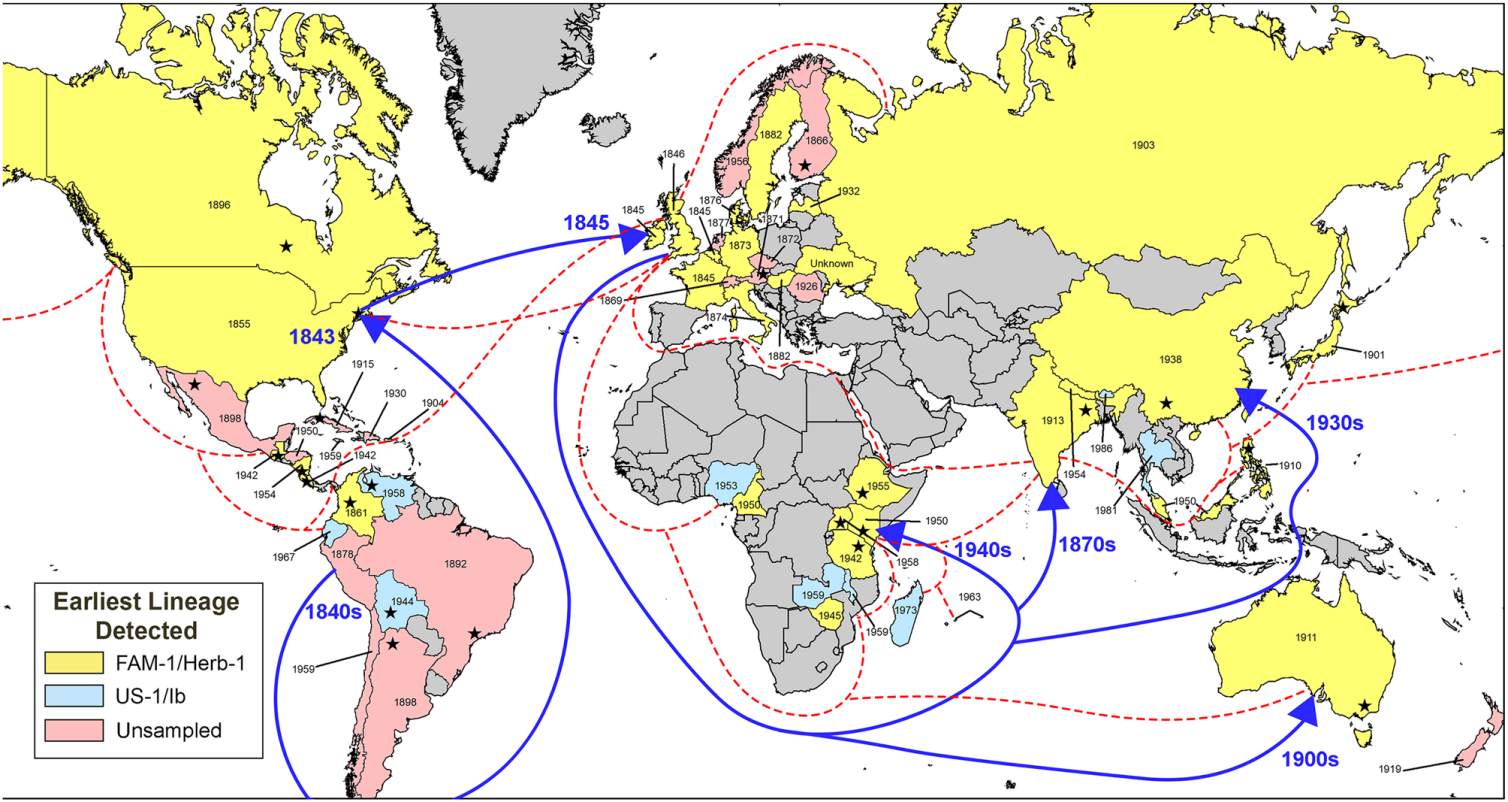
Global map of early outbreaks of late blight caused by *Phytophthora infestans.* Years within each country indicate the date of the earliest known specimen, while color indicates if the genotype was FAM-1, US-1 or unsampled. Dotted lines indicate representative trade routes of the British Empire circa 1932. Arrows indicate the most likely migration path taken by the FAM-1 lineage into Africa and Asia based on DIYABC analysis and trade routes. Stars within each country indicate the approximate location of the first recorded outbreak, if known. The map was generated using ArcMap 10.8 (http://desktop.arcgis.com).

A total of 137 historic samples were genotyped with microsatellites (Table 1). The FAM-1 genotype was the first lineage detected in specimens from most continents and countries sampled and was identified in 73% of the samples (Table 1) (Fig. 1). The US-1 genotype was identified later in 26% of the historic samples. A subset of FAM-1 samples (n = 67) were genotyped for mtDNA haplotype and all were identified as the Herb-1 (1a) mitochondrial haplotype, while all the US-1 lineages tested (n = 32) were the Ib mitochondrial haplotype.

**Table 1:** Summary population statistics of *Phytophthora infestans* populations from historic global outbreaks sorted by continent and genotype from herbarium specimens.

Population^a^		*N*	MLG	eMLG(SE)^c^	H	G	λ	Evenness	Hexp	Ia^d^	$\overline{r }$_*d*_
**Africa**	**Genotype**										
	FAM-1	5	5	5.00	1.61	5.0	1	1.000	0.437	0.936	0.1759
	US-1	13	13	10(7.3e−8)	2.56	13.0	0.9999	1.000	0.529	0.417	0.0571
	**Host**										
	*Solanum lycopersicum*	3	3	3	1.10	3.0	1	1.000	0.555	3.38	0.647
	*S. tuberosum*	13	13	10(7.30e−8)	2.56	13.0	0.9999	1.000	0.569	1.51	0.159
	Other (*Petunia *sp., *S. incanum*)	2	2	2	0.70	2.0	1	1.000	0.764	NA	NA
	Total	18	18	10(5.43e−7)	2.89	18.0	0.9995	1.000	0.568	1.80	0.191
**Asia**	**Genotype**										
	FAM-1	21	21	10.00	3.05	21.0	0.9996	1.000	0.404	0.136	0.0188
	US-1	11	11	10.00	2.40	11.0	0.9999	1.000	0.481	0.0458	0.00671
	**Host**										
	*S. lycopersicum*	4	4	4	1.39	4.0	1	1.000	0.561	3.25	0.411
	*S. tuberosum*	23	23	10(5.03e−7)	3.14	23.0	1.0005	1.000	0.497	1.08	0.111
	Other (*S. laciniatum*, *S. lyratum*, *S. marginatum*, *S. melongena*, *S. xanthocarpum*)	5	5	5	1.61	5.0	1	1.000	0.533	0.15	0.0261
	Total	32	32	10	3.47	32.0	1.0002	1.000	0.544	1.113	0.1168
**Europe**	**Genotype**										
	FAM-1	26	26	10.00(1.09e−6)	3.26	26.0	1.0005	1.000	0.390	0.274	0.0315
	US-1	3	3	3	1.10	3.0	1.0005	1.000	0.560	−1.11e−16	−8.33e−17
	**Host**										
	*S. lycopersicum*	3	3	3	1.10	3.0	1.334	1.000	0.386	−0.50	−0.50
	*S. tuberosum*	24	24	10(4.38e−7)	3.18	24.0	0.9997	1.000	0.506	2.77	0.26
	Other (*S. dulcamara*, *S. nigrum*)	2	2	2	0.70	2.0	1	1.000	0.548	NA	NA
	Total	29	29	10(1.03e−6)	3.37	29.0	1.0005	1.000	0.495	2.53	0.23
**North America**	**Genotype**										
	FAM-1	42	31	8.91(9.63e−1)	3.24	18.4	0.9691	0.711	0.337	−0.189	−0.0303
	US-1	6	6	6	1.79	6.0	0.9996	1.000	0.540	1.120	0.123
	**Host**										
	*S. lycopersicum*	3	3	3	1.10	3.0	1.0005	1.000	0.490	0.333	0.075
	*S. tuberosum*	43	43	10(9.46e−7e0)	3.76	43.0	0.9998	1.000	0.457	3.695	0.354
	Other (*S. nigrum*, *S. sp.*)	2	2	2	0.69	2.0	1	1.000	0.488	NA	NA
	Total	48	48	10(2.87e−6)	3.87	48.0	0.9998	NA	0.453	3.461	0.336
**Oceania**^**b**^	Total	2	2	2.00	0.70	2.0	1	1.000	0.286	NA	NA
**South America**	**Genotype**										
	FAM-1	5	5	5	1.61	5.0	1	1.000	0.480	2.289	0.2501
	US-1	3	3	3	1.10	3	1.0005	1.000	0.436	−0.333	−0.200
	**Host**										
	Other (*Petunia sp.*)	1	1	1	0.0	1.0	NA	NA	0.364	NA	NA
	*S. tuberosum*	7	7	7	1.95	7.0	0.9998	1.000	0.519	2.326	0.2204
	Total	8	8	8	2.08	8.0	0.875	1.000	0.566	0.982	0.0866
**Totals**	**Genotype**										
	FAM-1	101	85	32.7(1.57)	4.32	53.4	0.9908	0.718	0.381	0.41	0.0702
	US-1	36	36	36	3.58	36	0.9997	1.000	0.508	0.232	0.0258
	**Host**										
	*S. lycopersicum*	13	13	12	2.57	13.0	0.9999	1.000	0.461	1.45	0.194
	*S. tuberosum*	112	99	11.7(5.4e−1)	4.51	73.8	0.9950	0.813	0.461	2.12	0.220
	Other	12	12	12	2.48	12.0	1.0003	1.000	0.561	1.41	0.135
	Total	137	121	34(1.36e−0)^c^	4.69	82.7	0.9952	0.757	0.471	1.903	0.1952

^a^All isolates, including those with missing data (minimum = 5) are included.

^b^Only two samples were collected from Oceania, and both were collected from potato and identified as FAM-1.

^c^eMLG for the entire sample set was based on population counts by genotype.

^d^Data for Ia and $\overline{r }$_*d*_ were clone corrected before analysis.

*n* number of individuals, *MLG* number of multilocus genotypes (MLG), *eMLG* expected number of MLGs at smallest size of at least ten, *SE* standard error, *H* Shannon–Weiner Index of MLG diversity, *G* Stoddart and Taylor Index of MLG diversity, *λ* corrected Simpson’s Index, *Hexp* Nei’s 1978 gene diversity, *Ia* Index of Association, $\overline{r }$_*d*_ standardized index of association.

The earliest FAM-1 genotype was found in France in 1845 (K 71) and the most recent was found in Malaysia in 1987 (K 174) (Fig. 1, Supplementary Table 1). Surprisingly, this indicates that the famine lineage circulated for more than 144 years. In contrast, the earliest US-1 genotype was identified in a US specimen from 1931 (BPI 186927) and the most recent US-1 genotype was identified in a specimen from India from 1991 (IMI 344673). This indicates that the US-1 circulated for less than half the time of the FAM-1 lineage. Although US-1 has been found more recently and still occurs in parts of Africa^20^.

Greater subclonal variation was found in the FAM-1 than the US-1 genotype from all continents but Africa, where US-1 genotypes were more diverse (Table 1). There were 85 multilocus genotypes (MLGs) of FAM-1 and 36 MLGs of US-1 identified (Table 1). The greatest diversity of FAM-1 MLGs occurred in specimens from North America, Europe and Asia. FAM-1 displayed higher diversity values across all calculated indices including the Simpson’s index, which corrected for sample size. The FAM-1 also had a higher index of association (Ia) and standardized index of association ($\overline{\rm{r} }$_d)_ than US-1.

The majority of the herbarium samples studied (n = 112) were collected from potato, while only 13 were from tomato (Table 1). The remaining specimens were from petunia or wild species of *Solanum species* (n = 12). There were higher numbers of MLGs from potato than tomato, and greater genetic diversity and higher indices of association ($\overline{\rm{r} }$_d_) were found in specimens from potato than tomato.

Several SSR loci were useful for distinguishing the FAM-1 genotype from US-1. Diagnostic loci were Pi70 (192/192 in FAM-1 and 189/192 in US-1), PiG11 (160/200 in FAM-1 and 152/156/200 in US-1), PinfSSR2 (173/173 in FAM-1 and 173/177 in US-1), and Pi4B (209/213 in FAM-1 and 213/217 in US-1). The mean number of alleles was higher in FAM-1 (5.33) than in US-1(4.5) (Supplementary Table 2).

**Figure 2:**
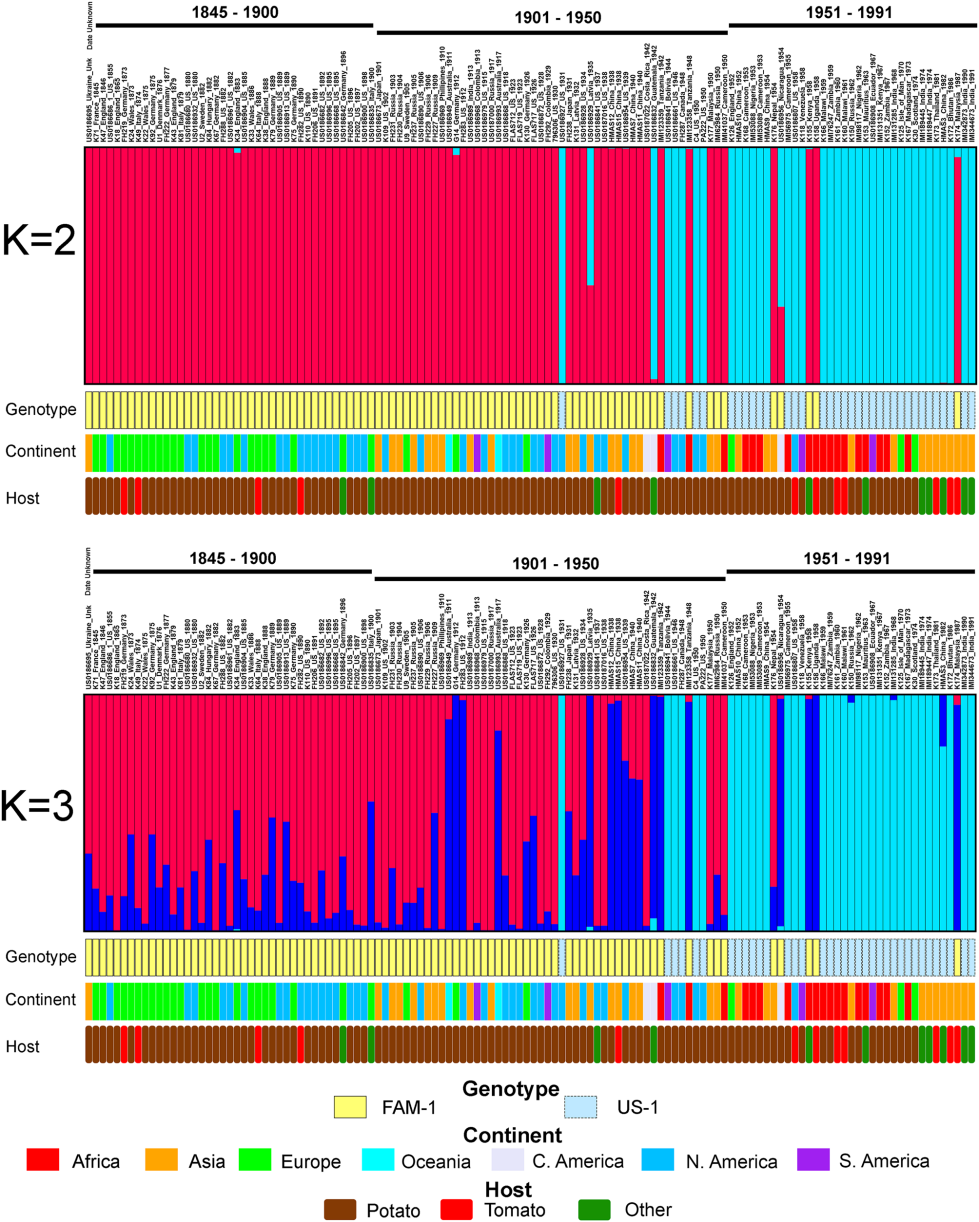
Structure analysis of microsatellite (SSR) genotypes of *Phytophthora infestans* from herbarium specimens. Specimens are arranged in ascending chronological order. Samples were clone corrected based on region before analyzing. Results displayed are based under the assumption of either two or three groups within the dataset (K = 2, K = 3). K = 2 was calculated to be the most likely based on the second order rate of change.

Structure analysis indicated the optimal K value was 2 based on results from Structure Harvester. At K = 2, the Structure analysis grouped samples into two groups based on genotype (FAM-1 or US-1). FAM-1 was found before US-1 on each continent (Fig. 2). However, at K = 3, US-1 genotypes were more homogeneous, while FAM-1 genotypes displayed allelic variation between the two remaining K groups. At K = 3, it was noted that FAM-1 genotypes showed more allelic diversity and shifted assignment from one K group to the other over time, beginning around 1911. Both FAM-1 and US-1 occurred in many geographic locations, but the earliest genotypes were found in the US and Europe.

**Figure 3:**
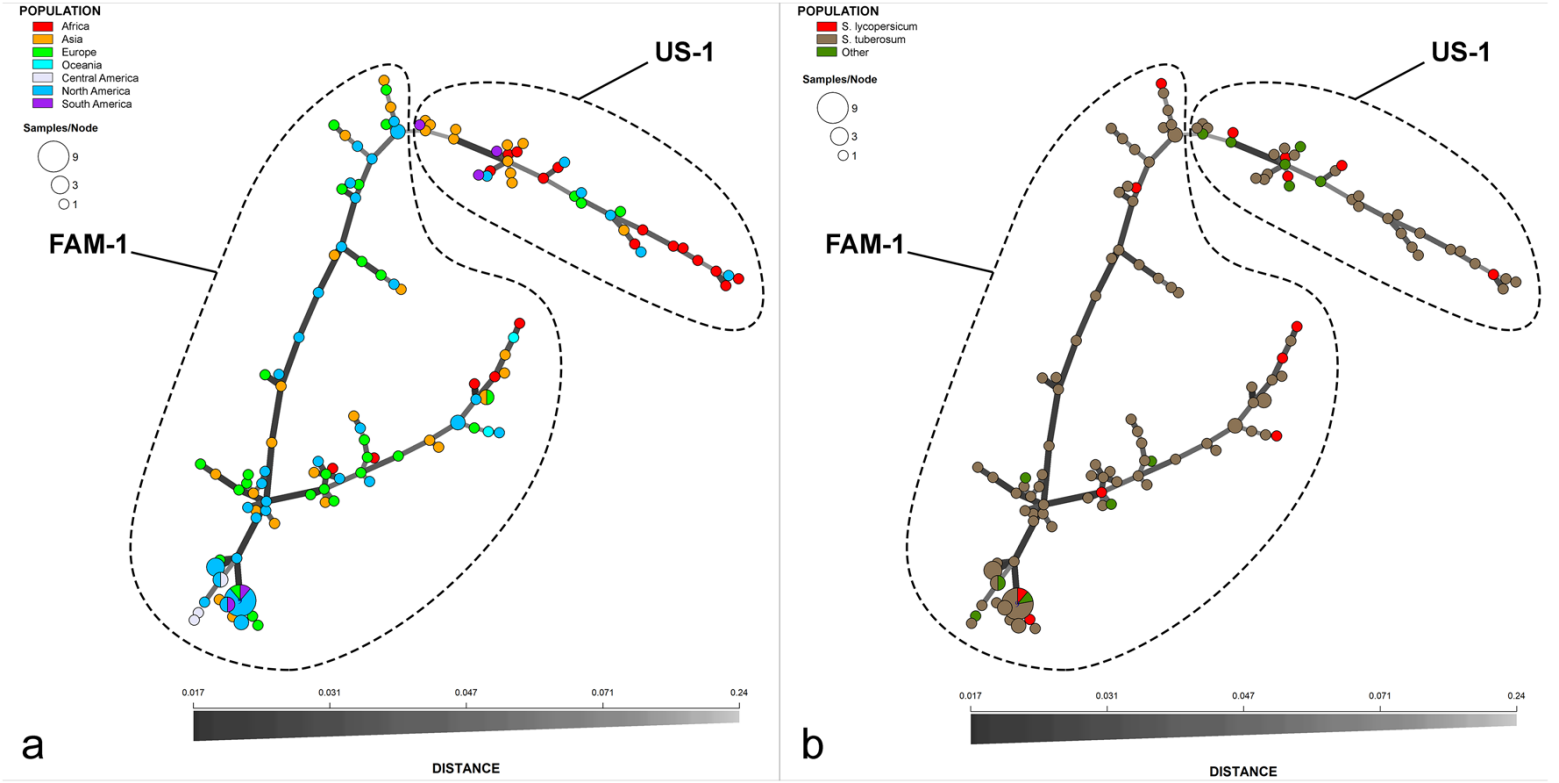
Minimum spanning network of SSR genotypes of *Phytophthora infestans* from herbarium specimens. Data are colored-coded based on (**a**) the continent where they were collected or (**b**) host. Genetic distance between haplotypes is indicated by the shade and thickness of the branches.

A minimum spanning network (MSN) was produced with haplotypes sorted by the continent where the samples were originally collected. Two large groups within the MSN consisting of either FAM-1 or US-1 MLGs were observed (Fig. 3). Within the clusters of genotypes, there was no genetic substructuring based on continent. However, FAM-1 had a larger number of MLGs and more branches in the MSN than the US-1 and greater subclonal variation was observed among FAM-1 MLGs (Table 1). No exclusive clusters were observed by country, host, or continent, but higher numbers of MLGs of FAM-1 were found in North America, Europe and Asia than elsewhere. There were fewer FAM-1 MLGs from Africa and they occurred on fewer branches of the MSN than MLGs from other continents, most likely due to the more recent introductions there. In contrast, there were more MLGs of US-1 in Africa than North America or Europe, indicating diversification of the US-1 MLGs in east Africa.

**Figure 4:**
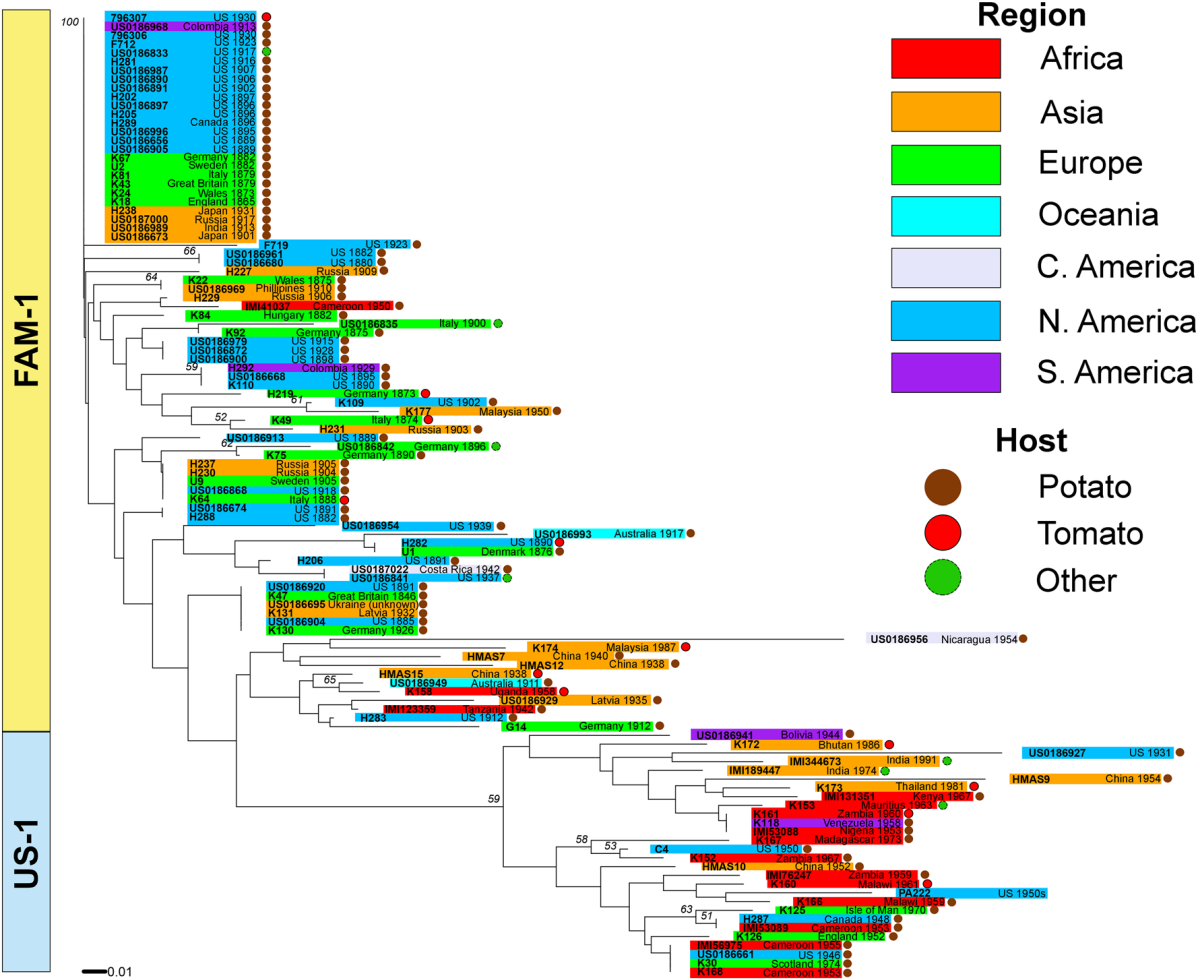
Neighbor joining tree of 7-plex microsatellite genotypes of *Phytophthora infestans* from herbarium specimens, Specimens are colored-coded based on the continent and host of sample. Bootstrapping was performed with 1000 replicates.

A similar structure was observed in the neighbor-joining tree developed using the SSR data, with two large clades that contained either FAM-1 or US-1 genotypes (Fig. 4). When the neighbor-joining tree was expanded to include modern global samples of *P. infestans*, most of the samples identified as FAM-1 or US-1 genotypes also formed distinct clusters within the larger neighbor-joining tree (Fig. S1).

### Migration

**Figure 5:**
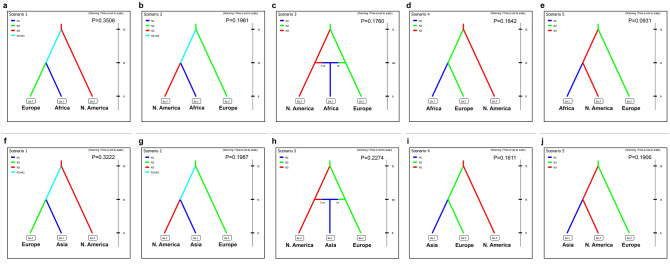
Posterior probabilities of migration scenarios involving populations of the FAM-1 genotype of *Phytophthora infestans* from North America and Europe to Africa **(A–E)** or North America and Europe to Asia **(F–J)**. Probabilities are based on 1% of the simulated data.

Potential migration paths of *P. infestans* into Africa and Asia were tested using specimens identified as FAM-1, as well as FAM-1 specimens from either Europe or North America. Central and South American samples were not included as source populations in migration analysis since there were not enough herbarium samples from those regions. We calculated the probabilities of scenarios that hypothesized movement of *P. infestans* based on a North American source, a European source, and either source with a constant or a varying population size, and a source based on an admixture of European and North American populations (Fig. 5).

The most likely scenario indicated a divergence first of the European genotypes of FAM-1 from a North American source, then the divergence of either the African or Asian FAM-1 lineages from the European source (Fig. 5a,f). Scenario 1, P = 0.3506 and 0.3222 for Africa and Asia, respectively) (Supplementary Table 3). Confidence in the scenario choice was evaluated by using simulated datasets to calculate error percentages between the three scenarios with the highest probabilities. Estimation of type I error for scenarios using the Asian data revealed that 53.2% of simulated datasets using this scenario resulted in the highest posterior probability for Scenario 1 when compared to the two scenarios with the next highest probabilities (Scenarios 3, 2) (type I error, 0.468) (Fig. 5b,c and Supplementary Table 3) or the others (Fig. 5d,e). For scenarios using the African data, estimation of type I error indicated that 35.6% of simulated datasets resulted in the highest posterior probability for Scenario 1 (Fig. 5f) when compared to the next highest probabilities (Scenarios 2, 4) (type I error, 0.644) (Fig. 5g,i) or the rest (Fig. 5h,j).

## Discussion

We examined historic outbreaks of *P. infestans* from global historic sources to better understand the history of the spread of the pathogen after the first recorded outbreaks in Europe and the US. The FAM-1 genotype survived over 140 years and was found on six continents. Our data revealed the widespread presence of the FAM-1 genotype throughout the world and its dominance until the 1930s, when the US-1 lineage began to emerge globally. By the end of the 1950s, FAM-1 had almost completely disappeared and was displaced by the US-1 genotype, most likely through movement of potatoes for resistance breeding^2,4,10^. The only post-1950s FAM-1 genotype observed was collected in a sample from Malaysia in 1987. This unusual sample suggests that the FAM-1 genotype may have continued to persist in remote areas of the world for a longer period. The Malaysian FAM-1 genotype had variable alleles at several loci when compared to earlier FAM-1 genotypes, suggesting the accumulation of mutations over time or potential recombination with another lineage such as US-1. The Malaysian sample of FAM-1 clustered closely with the US-1 genotype in a neighbor-joining tree of a larger set of samples. Further work is underway to sequence the genome of this specimen to understand the genomic variation in more detail.

The earliest known record of the US-1 genotype in potato is from 1931 in the US^10^. Based on specimens analyzed in this study, the earliest known records of US-1 in Africa are from 1953 in Cameroon and Nigeria. For Asia, the oldest US-1 sample observed in this study was from 1952 in China. In South America, the oldest US-1 lineage was from Bolivia in 1944. US-1 was not identified from Australia/Oceania in this study, although that may be due to the minimal number of specimens available in the collections we sampled. Further work is underway in our lab to genotype more historic specimens from the New South Wales Mycological Herbarium (DAR) in Australia.

There is scarce information on the history of the early emergence of US-1, but records from the literature suggest an approximate period for multiple parts of the world. In 1947, while documenting the history of late blight in Tasmania, Oldaker commented on an unusual outbreak of *P. infestans* in 1938. The disease was more sporadic than it had been in previous years, but treatment with copper formulations proved effective in controlling the pathogen^18^. In 1951, Nattrass wrote that potatoes bred for *P. infestans* resistance were failing with the emergence of a new biotype that appeared in Tanzania in 1946^19^. Our data suggest the new biotype observed was likely the US-1 lineage in Africa. The proximity of these outbreaks suggests that US-1 genotype was spread during British potato breeding trials, facilitated by the continued movement of tubers over long distances into the continent.

Both the FAM-1 and US-1 genotype predominantly formed two clusters that excluded all modern lineages from Europe, North America, and South America. This indicated that these genotypes were distinct from modern lineages and most likely migrated from a different source^10^. Data from whole genome sequences indicate that FAM-1 is ancestral to other modern circulating *P. infestans* lineages and shares ancestry with *P. andina* in South America^11^. Both genotypes likely migrated into the US from an Andean source^10,11,14^. However, previous studies of North American populations of *P. infestans* suggest a Mexican origin of other recent aggressive lineages of *P. infestans* circulating in the US^14,15^. It is important to note that FAM-1 and US-1 were not identified in any Mexican samples we tested, however few herbarium specimens exist from this country or countries in South America. FAM-1 was found in the oldest South American samples from Colombia, which coincide with the oldest published reports of the disease that are from this country ^21–23^.

Migration analyses of FAM-1 genotype was analyzed using DIYABC analysis and data suggest that both the African and Asian genotypes of FAM-1 most likely emerged from a European source. Outbreaks of the disease caused by FAM-1 genotype first occurred in North America and subsequently spread 2 years later to Europe^2,3^^;^^10^. This coincides with historic records and our previous studies that support the migration of *P. infestans* into Europe after outbreaks occurred in North America^2,4,6,10,22^. The top migration scenario for emergence of *P. infestans* into Africa and Asia is from a European source. We do not attempt to identify source countries within Europe but likely sources from historical records include countries such as Great Britain and/or the Netherlands, which exported potatoes into both continents during colonization.

Our data and examination of global herbarium sources suggest that the pathogen likely moved first on potatoes and then spread later into tomato^22^. There was greater genetic diversity among potato than tomato genotypes of FAM-1 and more MLGs among the potato genotypes. In addition, spread in ripe tomato fruit would have been unlikely in nineteenth century ship holds. Potato tubers would have survived global journeys in steam ships^2–4,22^.

The findings of our study are supported by historical reports published by researchers contemporary to the time of the initial *P. infestans* outbreaks on various continents and provide insight into potential sources^3,4,17–19,23^. Potatoes were disseminated across the world by European sailors and missionaries, with varying degrees of adoption by local populations^24,25^. As European colonists moved into new regions, potatoes moved with them. Potatoes were actively encouraged as food for local populations during colonization and touted as cheap and nutritious. In India, potatoes were included among foods that intended to “increase happiness and improve the well-being of India’s rural population”, and were subsequently used as food for the labor force of the colonizing empire^26^. This mentality resulted in the dissemination of potatoes to local villages by horticultural and agricultural societies, despite the crop having already been adopted as a cash crop to sell to British soldiers^26,27^.

Potatoes were shipped into colonial regions long after the establishment of the crop. A colonial handbook for Kenya from 1920 states that potatoes grown from locally produced seed were not as productive, and recommended regularly importing fresh seed potatoes from Europe^28^. This would have provided an obvious avenue for the introduction of new genotypes of *P. infestans*. Regular imports of potatoes were observed in other parts of the world as well. A 1904 agricultural report for the Philippines compared the quality of locally grown potatoes to imported ones found in Manila markets, suggesting a potential introduction route, most likely via the US^29^. In India, the pathogen was reported in the area circa 1870–1880 based on reports from local agri-horticultural societies^17^. In letters it was stated that a major late blight outbreak in the Nilgiri region around 1893 was the result of the importation of potatoes from a large nursery in England^17^. In East Africa it was believed that the first outbreak located outside of Nairobi, Kenya, was the result of an importation of Kerr’s Pink potatoes for planting from England, bolstered by a wet and rainy season^4^. In West Africa, however, it was thought that *P. infestans* was introduced as the result of the importation of potatoes from France. While intended for use as food, potatoes were also planted, resulting in the propagation of the pathogen^24^. *P. infestans* followed the movements of colonists through potatoes, leading to its introduction from the US first into Europe and subsequently into the African, Asian and the Australian/Oceanic continents.

With the extensive reach of the British Empire (Fig. 1), it is likely that many introductions of the pathogen were the result of movement of potatoes on British ships, with multiple introductions over time as new shipments of tubers were imported into British colonies. The FAM-1 genotype was diploid^11^ and asexual and was able to colonize susceptible potatoes on six continents and thus caused global pandemics. It is clear that the FAM-1 genotype adapted to many environments, occurred mostly on potato, and remained aggressive for more than 140 years. Unlike modern lineages of *P. infestans* that change rapidly^2^ due to sexual recombination or deployment of potatoes with new resistance genes, FAM-1 remained virulent and survived for almost a century and a half.

## Methods

Over 1280 late blight specimens collected in the nineteenth and twentieth century are in herbaria on six continents^22^. We sampled specimens from 37 countries on six continents including North America, South America, Europe, Africa, Asia and Oceania (Fig. 1) (Supplementary Table 1). A total of 137 samples were genotyped with microsatellites. *Phytophthora infestans* was sampled from herbarium specimens collected in Africa, Asia, Europe, Oceania, North America, Central and South America. A total of 137 samples were analyzed, consisting of 18 African samples (1942–1973), 32 Asian samples (1901–1991), 2 Oceania samples (1911–1917), 29 European samples (1873–1970), 48 North American samples (1855–1958), and 8 South American samples (1913–1967) (Supplementary Table 1).

We collected SSR data for an additional 194 samples from modern collections, databases, published studies, and theses^10,30,31^. These included data from modern populations^10^ and representatives of recent common European lineages from Euroblight.net. In addition, we included a subset of data from publications on recent Mexican populations^30,31^.

### DNA extraction, PCR, and genotyping

DNA was extracted from lesions present on each herbarium specimens using either a Qiagen DNEasy Plant Mini Kit (Qiagen, Valencia, CA) or a modified CTAB method using DNEasy Plant Mini Kit spin columns for cleaning and purifying DNA^12^. The presence of *P. infestans* DNA was checked using species specific primers^32^. All work with herbarium DNA was conducted in a containment lab in which no modern DNA of *P. infestans* is used, using separate equipment and reagents.

Mitochondrial haplotyping of samples was conducted using primers and PCR cycling conditions developed by Griffith and Shaw to detect the presence of the Ib haplotype^11,33^. For detecting the Herb-1 haplotype we utilized primers previously developed that target a single nucleotide polymorphism within the haplotype^7,10^. Amplicons were sequenced at the Genomic Sciences Laboratory at North Carolina State University.

Samples were genotyped using a 12-plex system of primers for the identification of *P. infestans* lineages using microsatellite loci^34^. To compensate for the low levels of DNA present in extractions, a modification of the PCR protocol was used that increased primer concentration, sample size, and cycling times. The Qiagen Type-It Microsatellite PCR kit (Qiagen Corporation, Valenica CA) was used for PCR reactions, and sample volumes were modified to run a 12.5 µL reaction, consisting of 6.25 µl of Type-It 2× master mix, 1.3 µl of a 10× primer mix (Supplementary Table 4), 1.95 µl ddH2O, and 1–3 µl of DNA extract. Thermal cycling conditions consisted of initial denaturation at 95 ºC for 5 min, followed by 33 cycles of 95 ºC for 30 s, 58 ºC for 90 s, and 72 ºC for 30 s, and then a final extension period for 30 min at 60 ºC. Fragments were analyzed on an Applied Biosystems 3730xl DNA analyzer at the Genomic Sciences Laboratory at North Carolina State University using 1–3 µl of PCR product in a 10.3 µL reaction mix consisting of 10 µL highly deionized formamide and 0.3 µL LIZ500 size standard (Applied Biosystems, Foster City, CA). Alleles were scored in Geneious 11.1.5 (Biomatters Ltd., Auckland, NZ) using microsatellite plugin 1.4.6. Alleles were named using bin ranges from previously published work ^34,35^.

### SSR data analysis

Because of the age of herbarium DNA, recovery rates of microsatellite loci from *P. infestans* are lower than they would be for DNA extracted from modern samples, resulting in increased missing data. To reduce the amount of variability due to missing data, only samples with data from at least 5 SSR loci were used for analysis. Data were divided into categories based on continent: Africa (Afr), Asia (As), Europe (EU), North America (NA), Oceania (Oc), and Central and South America (SA). The broad structure of the populations was evaluated via model-based Bayesian clustering using the program Structure v. 2.3.3^36^. Before analysis by Structure, the data were clone corrected (clones were removed such that each population contains only one representative of each haplotype) using the R library poppr v. 2.8.1^37^ and R v. 3.5.2^38^. Data were clone corrected using their region of collection as a population. The data were run using a 20,000 repeat burn-in and 1,000,000 MCMC repeats under a no admixture model, with each individual sample representing its own population. Independent runs of the model used *K* values from 1 to 10 with 10 replicate runs at each value of *K*. The optimal *K* was estimated using the second order rate of change (the “Evanno method”) in the web tool Structure Harvester^39,40^. All runs for the optimal *K* value, as well as non-optimal *K* values, were averaged using CLUMPP v. 1.1.2^41^ using the Greedy algorithm (M = 2) with the pairwise matrix similarity statistic G’ (S = 2). The Greedy algorithm was used with 1000 repeats of randomly selected input orders. The resulting output was visualized with the program distruct v. 1.1^42^.

Poppr was also used to infer population statistics including the number of samples (N), the number of MLGs, the number of expected MLGs at the smallest sample size of at least 10 (eMLG)^43^, the Shannon-Weiner index of MLG diversity (H)^44^, the Stoddart and Taylor index of MLG diversity (G)^45^, Simpson index corrected for sample size by multiplying the index value by N/(N-1) (λ)^31,46^, evenness^47–49^, Nei’s unbiased gene diversity (Hexp)^50^, the index of association (Ia)^51,52^, and the standardized index of association($\overline{r }$_*d*_)^53^.

Relationships between locations and haplotypes of samples were further explored using a minimum spanning network (MSN) based on Bruvo’s distance using the R library adegenet v 2.1.1^54^. In addition, a neighbor-joining (NJ) tree based on Bruvo’s distance was constructed using the poppr R library and a combination (genome addition and genome loss) model. In order to utilize a complete dataset in the NJ tree for the purposes of bootstrapping, five loci with low recovery rates were removed for tree construction (PinfSSR8, PinfSSR4, Pi63, PinfSSR11, Pi4B) to create a 7-plex dataset (Fig. S1). Any remaining samples still containing missing data were removed. The tree was bootstrapped using 1000 samplings.

An additional 7-plex neighbor-joining tree was generated utilizing the trimming method described above and using the combined dataset of herbarium and modern samples. Due to the bootstrapping function used (bruvo.boot), no samples with missing data or null alleles could be used. Therefore, samples with putative null alleles were also removed from the dataset for the neighbor-joining tree.

### Migration of FAM-1

Migration routes of the FAM-1 lineage of *P. infestans* into Africa and Asia from Europe and/or North America were examined using Approximate Bayesian Comparison (ABC), as implemented in the program DIYABC v. 2.0.4^55^. Tested migration scenarios for both African and Asian populations included direct divergence from Europe or North America, divergence including a change in population size, or admixture between European and North American populations. Parameter range priors were initialized with values from Saville et al.^10^ and then iteratively modified to better fit our data (Supplementary Table 5). A total of 5 million datasets were simulated. Scenario probabilities were determined through comparison of the observed dataset to simulated datasets generated by DIYABC. A logistic regression of these differences was computed using ten proportions of the simulated dataset as the dependent variable and corresponding differences between the observed and simulated datasets as the independent variable. The value calculated using 50,000 simulated datasets was taken as the scenario’s overall probability. Confidence in the highest scenario was evaluated using a type I error test, in which the data were compared against 500 simulated data sets assuming the scenario with the highest probability is true and the number of times the scenario in question was correctly or incorrectly applied to the data was determined.

## Supplementary Information

Supplementary Information.

## Data Availability

Raw SSR data and binning rules can be found in GitHub (https://github.ncsu.edu/acsavill/Pinf-herbarium-datasets). See the references in the Supplementary Information for data used in the analysis.
